# Pharma Websites and “Professionals-Only” Information: The Implications for Patient Trust and Autonomy

**DOI:** 10.2196/jmir.7164

**Published:** 2017-05-24

**Authors:** Mark Alan Graber, Eliyakim Hershkop, Rachel Ilana Graber

**Affiliations:** ^1^ Departments of Emergeny and Family Medicine University of Iowa Carver College of Medicine Iowa CIty, IA United States; ^2^ Medical School Technion Israel Institute of Technology Hiafa Israel; ^3^ Unaffiliated Washington DC, DC United States

**Keywords:** trust, ethics, personal autonomy, readability

## Abstract

**Background:**

Access to information is critical to a patient’s valid exercise of autonomy. One increasingly important source of medical information is the Internet. Individuals often turn to drug company (“pharma”) websites to look for drug information.

**Objective:**

The objective of this study was to determine whether there is information on pharma websites that is embargoed: Is there information that is hidden from the patient unless she attests to being a health care provider? We discuss the implications of our findings for health care ethics.

**Methods:**

We reviewed a convenience sample of 40 pharma websites for “professionals-only” areas and determined whether access to those areas was restricted, requiring attestation that the user is a health care professional in the United States.

**Results:**

Of the 40 websites reviewed, 38 had information that was labeled for health care professionals-only. Of these, 24 required the user to certify their status as a health care provider before they were able to access this “hidden” information.

**Conclusions:**

Many pharma websites include information in a “professionals-only” section. Of these, the majority require attestation that the user is a health care professional before they can access the information. This leaves patients with two bad choices: (1) not accessing the information or (2) lying about being a health care professional. Both of these outcomes are unacceptable. In the first instance, the patient’s access to information is limited, potentially impairing their health and their ability to make reasonable and well-informed decisions. In the second instance, they may be induced to lie in a medical setting. “Teaching” patients to lie may have adverse consequences for the provider-patient relationship.

## Introduction

About 67% of physician office visits involve a prescription medication [1]. This rises to 80% when the visit is to an emergency department [1]. Ideally, the provider should discuss the prescriptions’ benefits and side effects with the patient. However, the scheduling realities of single visits restrict patient contact time and the information that can be presented. Unfortunately, providers often do not discuss medication side effects, and so on with patients [2,3].

Inevitably, and reasonably, patients seek out additional information. One source of this information is the Internet, including pharma websites. It is not an exaggeration to say that the use of the Internet to search for medical information is ubiquitous. In 2012, 72% of Internet users looked for medical information on the Web, and this percent has been increasing with time [4-6]. Whereas the quality of Web-based information varies greatly, unfettered access to information on the Internet is critical to patients trying to educate themselves. In order to make a truly autonomous decision, patients must have whatever information they feel is necessary. Whereas providers may not have the time to review all of the information a patient may want to know, searching the Internet is not subject to a time limitation.

The purpose of this study was to investigate a convenience selection of pharma websites in order to determine whether there is information labeled as for “professionals-only” (eg, not for patients), and what one needs to do in order to access this information. We discuss our findings within the ethical framework of Western medicine, including respect for autonomy and truth telling.

## Methods

We examined a convenience sample of 40 pharma websites looking for the presence of a “professionals-only” section. Drugs websites to review were selected based on the year of approval by the Food and Drug Administration (FDA; within the past 3 years), as well as a sample of commonly prescribed medications. There was no attempt to randomize the selection. All of the researchers reviewed each site to determine whether (1) a “professionals-only” section exists, and (2) whether access to this information requires certifying that one is a health care professional.

This project did not require an institutional review board (IRB) submission because no human (or animal) subjects were involved. As noted in the University of Iowa IRB Statement of Compliance, “The University of Iowa Institutional Review Boards are duly constituted with written procedures for initial and continuing review of human subjects research” [7]. This research does not include any human subjects.

## Results

**Figure 1:**
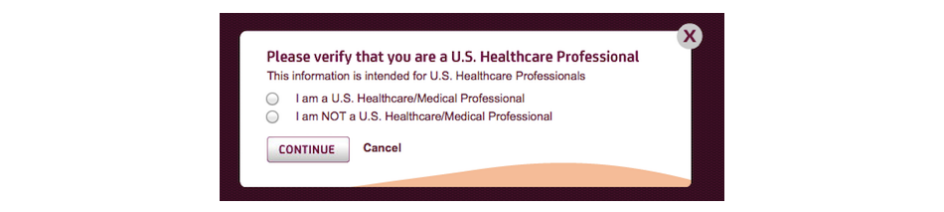
An example of a “professionals only” attestation statement from a drug company website.

**Figure 2:**
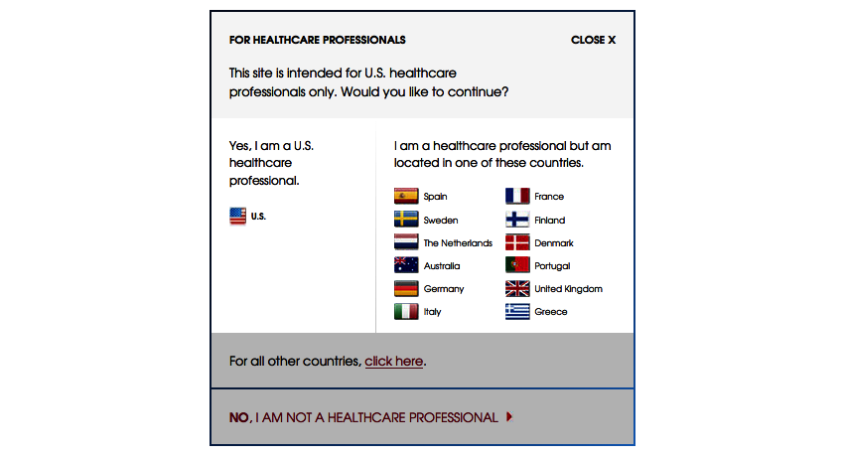
An example of a “professionals only” attestation statement from a drug company website.

Of the 40 websites, 36 (90%) contained information or a link to information that was restricted to professionals only (Table 1). Twenty-four (67%, 24/36) of these 36 sites required an attestation that the user was a US health care professional before the information could be accessed (Table 1). Another 12 (33%, 12/36) of the 36 contained “professionals-only” information that was accessible without such an attestation. Figures 1 and 2 are screenshots that show of the type of verification required in order to access “professionals only” information. Researchers agreed on the categorization of all sites.

**Table 1:** Results of website evaluation.

Trade name	Drug name	Website	Embargoed information	Comments
Abilify	Aripiprazole	www.abilify.com	Yes^a^	
Amyvid	Florbetapir F18 injection	www.amyvid.com	Yes	
Anoro Ellipta	Umeclidinium and vilanterol inhalation powder	www.myanoro.com	Yes	
Briinta	Ticagrelor	www.briinta.com	Other^b^	
Brintellix	Vortioxetine	www.brintellix.com	Other	
Corlanor	Ivabradine	www.corlanor.com	Yes	
Cometriq	Cabozantinib	www.cometriq.com	Yes	
Crestor	Rosuvastatin	www.crestor.com	Yes	
Cyramza	Ramucirumab injection	www.cyramzahcp.com	Yes	
Dexilant	Dexlansoparzole	www.dexilant.com	Other	
Duavee	Conjugated estrogens or bazedoxifene	www.duavee.com	Other	
Eliquis	Apixiban	www.eliquis.com/eliquis	Yes	
Entresto	Sacubitril or valasartan	www.entresto.com	Other	
Erivedge	Vismodegib	www.erivedge.com/	Yes	
Farxiga	Dapagliflozin	www.farxiga.com	Yes	
Focalin XR	Dexmethylphenidate	www.focalinxr.com	No^c^	
Gazyva	Obinutuzumab	www.gazyva.com	Yes	
Invokana	Canagliflozon tablets	www.invokana.com	Yes	
Harvoni	Edipasvir and sofosbuvir	www.harvoni.com	Yes	
Kadcyla	Ado-trastuzumab emtansine	www.kadcyla.com	Yes	
Kalydeco	Ivacaftor	www.kalydeco.com	Yes	
Kynamro	Mipomersen sodium	www.kynamro.com	Yes	Requires sign-up
Lipitor	Atorvastatin	www.lipitor.com	No	
Myalept	Metreleptin for injection	www.myalept.com	Yes	
Nesina	Alogliptin	www.nesinahcp.com	Other	
Otezla	Apremilast	www.otelza.com	Other	
Picato	Ingenol mebutate	www.picato.com	Yes	
Pomalyst	Pomalidomide 29	www.pomalyst.com	Yes	
Pradaxa	Dabigitran	www.dabigitran.com	Yes	
Praluent	Alirocumab	www.praluent.com	Yes	
Pristiq	Desvenlofaxine	www.pristiq.com	Other	
Savaysa	Edoxaban	www.savaysa.com	Other	
Sovaldi	Sofosbuvir	www.sovaldi.com	Other	
Stribild	Elvitegravir, cobicistat, emtricitabine, tenofovir, and disoproxil	www.stribild.com	Other	
Tanzeum	Albiglutide	www.tanzeum.com	Yes	
Tecfidera	Dimethyl fumarate	www.tecidera.com	Yes	
Voraxaze	Glucarpidase	www.voraxze.com	Yes	
Vraylar	Cariprazine	www.vraylar.com	Yes	
Vyvanse	Lisdexamfetamine	www.vyvanse.com	Other	
Xarelto	Rivoroxiban	www.xarelto.com	Other	

^a^Yes = embargoed “professionals-only” information requiring attestation.

^b^Other = professionals-only information but no attestation required.

^c^No = no “professionals-only” information.

## Discussion

### Principal Findings

Restricting access to information as implemented by these websites has several implications. In the first instance, patients may not have access to desired information. Anything that limits a patient’s knowledge has the potential to adversely impact autonomy [8,9]. It can be argued that patient-oriented pharma websites contain complete information and already present all of the information that a patient needs in a useable form. Whereas it might be intuited that this solves the problem of information access, patient-oriented pharma websites may present a skewed view of drug risks and benefits and often contain advertising designed to entice the patient [10-12]. One might also argue that the lack of access to “professionals-only” information can be mitigated by the US FDA (the agency responsible for the approval and safety of drug in the United States) having made it a requirement that pharma advertising allow patient access to the package insert, including Web-based advertising [13]. However, the language of packaging information (and medical information on the Web in general) is written at a level that is often incomprehensible to the general public [14-20]. Thus, providing unfettered access to the “professionals-only” information will not in itself solve the problem of lack of accessibility; enhanced readability and a better presentation of information would generally be needed for the information consuming public. This is not an unsurmountable barrier; difficult information can be presented in a manner that is accessible to the general public [19]. We can enhance individuals’ ability to make autonomous choices by improving the quality of information presentation.

One can argue that consumers can get the same information elsewhere on the Internet (eg, wikis, sites such as drugs.com). This creates a fundamental dilemma, however. For patients to know what information is missing from their current knowledge base, they first need to access the “professionals-only” information. Thus, the argument that the same information is available elsewhere fails. This may be the case but the uncertainty in patients’ minds will continue.

A second, and perhaps more troubling, dilemma arises when there is “professional-only” information on pharma websites that requires an attestation as to one’s status as a health care provider. The patient can then either (1) not access the information or (2) lie about their status as a professional. Either outcome is unacceptable. Introducing lying into the therapeutic space can have adverse consequents. Requiring patients to lie in order to obtain information sets a bad precedent. If lying is tacitly accepted as part of the medical system, it has the potential to undermine one of the pillars on which medicine is built; truth telling. Worse still, the system that a patient would like to have faith in is enticing them to lie. Forcing patients to lie may challenge one’s faith in the truthfulness of other aspects of care and the medical system [21,22].

Finally, “professionals-only” information can suggest that health care professionals have some dark, mysterious secrets to be hidden from the public. Patients might legitimately wonder what it is that we health care professionals have to hide. Such concerns might further undermine trust in the medical system. Such distrust, whether generated by the perception that there is information hidden from the patient or because of the requirement to lie in the medical sphere, translates into worse patient outcomes [23,24].

It is true that patients can become overwhelmed with information. One solution is to have providers act as interpreters of information; this is the model preferred by many patients [21]. This puts the onus on providers; unfortunately, providers often do not do a particularly good job of laying out the benefits and harms of therapy either [25]. However, a provider-patient partnership with the provider interpreting information for the patient and directing the patient to reliable Web-based information can be a viable model [26].

### Limitations

We did not look at the content of “professionals-only” websites and compare this with the content of the patient-oriented websites. For the purposes of our study, such a comparison is a secondary consideration and would not change the study outcome; our main purpose here is to highlight the ethical issues surrounding “professionals-only” websites needing an attestation. A follow-up study could be designed to examine the issue of content. However, no matter the outcome of such an analysis, the basic dilemma remains; patients have to lie to get to “professionals-only” content or do without this information. If the content of the patient and the professional is the same, why require an attestation which may prompt the patient to lie? If the content of the professionals-only and patient-only websites are different, why are we not providing patients with the same information that we are providing to professionals (albeit presented in a manner appropriate for lay-people)?

A second weakness of our study is that we only looked at a small sample of pharma websites. It is possible, but unlikely, that many other pharma websites do not have embargoed information. Nonetheless, we have shown that there is a potential problem related to embargoed information, lying, and trust.

### Conclusions

In conclusion, pharma websites often have a “professionals-only” section where access requires user attestation that she is a health care provider. Three problems follow. First, limited access potentially restricts patient information and therefore autonomy. Second, nonaccess may suggest that professionals have “something to hide.” Finally, such a system may “train” or induce patients to lie in other medical interactions. Distrust of the medical system may ensue.

## Abbreviations

US FDA: United States Food and Drug Administration
